# Establishing a gold standard for manual cough counting: video versus digital audio recordings

**DOI:** 10.1186/1745-9974-2-6

**Published:** 2006-08-03

**Authors:** Jaclyn A Smith, John E Earis, Ashley A Woodcock

**Affiliations:** 1 North West Lung Research Centre, South Manchester University Hospitals Trust, Wythenshawe Hospital, Southmoor Rd, Manchester, M23 9LT, UK; 2 University Hospital Aintree, Longmoor Lane, Liverpool, Merseyside, L9 7AL, UK

## Abstract

**Background:**

Manual cough counting is time-consuming and laborious; however it is the standard to which automated cough monitoring devices must be compared. We have compared manual cough counting from video recordings with manual cough counting from digital audio recordings.

**Methods:**

We studied 8 patients with chronic cough, overnight in laboratory conditions (diagnoses were 5 asthma, 1 rhinitis, 1 gastro-oesophageal reflux disease and 1 idiopathic cough). Coughs were recorded simultaneously using a video camera with infrared lighting and digital sound recording.

The numbers of coughs in each 8 hour recording were counted manually, by a trained observer, in real time from the video recordings and using audio-editing software from the digital sound recordings.

**Results:**

The median cough frequency was 17.8 (IQR 5.9–28.7) cough sounds per hour in the video recordings and 17.7 (6.0–29.4) coughs per hour in the digital sound recordings. There was excellent agreement between the video and digital audio cough rates; mean difference of -0.3 coughs per hour (SD ± 0.6), 95% limits of agreement -1.5 to +0.9 coughs per hour. Video recordings had poorer sound quality even in controlled conditions and can only be analysed in real time (8 hours per recording). Digital sound recordings required 2–4 hours of analysis per recording.

**Conclusion:**

Manual counting of cough sounds from digital audio recordings has excellent agreement with simultaneous video recordings in laboratory conditions. We suggest that ambulatory digital audio recording is therefore ideal for validating future cough monitoring devices, as this as this can be performed in the patients own environment.

## Background

For more than 40 years there has been an interest in making objective measurements of cough frequency. The original published systems consisted of reel-to-reel tape recorders with patients confined to a single room containing a microphone [1-3]. Coughs were manually counted by listening to the sound recordings. The major problems with these systems were the laborious nature of the manual cough counting and the restriction of the patients; hence these static systems never became established.

In the 1990s ambulatory devices using analogue sound recordings combined with EMG were devised; coughs were identified manually from the visualisation of the waveforms [4,5]. Validation of these devices was limited to simultaneous non-ambulatory sound recordings over short periods of time, as the devices waveforms could not be listened to, to check their identity.

In order to make cough monitoring applicable to clinical practice, it is necessary to develop accurate automatic detection and counting of coughs. Automated devices would make large studies feasible and may allow endpoints other than cough counts to be measured e.g. amplitude, temporal pattern and cough sound quality.

Although an acceptable automated cough monitoring system is not yet available this area continues to progress [6-11]. With the availability of digital recording devices and the advances in digital storage media, battery powered mp3 player/recorders can be used to make high quality ambulatory sound recordings. These enable cough to be recorded in a patient's home environment. Data can be transferred to personal computer and the recordings used to develop algorithms to identify cough sounds. The question still remains as to the best method to validate any new system.

Previous studies have used video recordings with real-time manual counting of cough as the gold standard [12,13]. The advantage of using video for cough detection is the visualisation of the subjects' movements as well as hearing the characteristic sound can be used to verify cough events. The two main disadvantages are the lengthy process of reviewing the recorded material and the limited field of vision of the camera, restricting the subjects' activities.

The aim of this study was to establish whether video in addition to audio recording was necessary to accurately manually count coughs and hence provide a gold standard for validation of novel cough monitoring systems. We performed simultaneous overnight video and digital audio recordings, in patients complaining of chronic cough and compared the manual cough counts from each media.

## Methods

### Subjects

Eight patients with chronic cough were recruited from the out-patients department of the North West Lung Centre. Simultaneous overnight cough recordings using digital audio and video were made in laboratory conditions. Approval was obtained from the local research ethics committee and all subjects gave written consent.

### Quantification of cough

**Figure 1:**
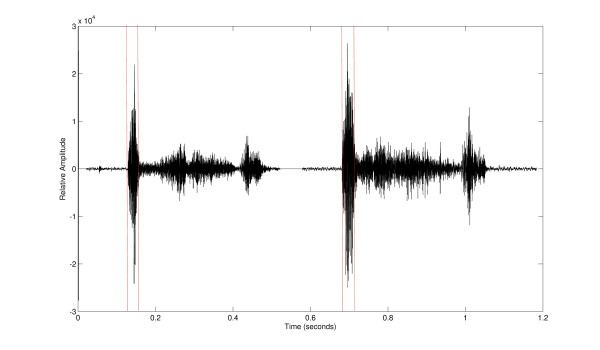
Two coughs with the explosive phase of the cough sounds marked by the vertical dashed red lines.

Cough sounds were manually counted by a single trained observer. The order in which the recordings were counted for each individual (digital or video) was randomly allocated. Coughs were quantified by counting the number of explosive cough phases (see Figure 1). The explosive phase is always present in a cough sound and is *the *characteristic sound we recognise as cough. In a peel of coughs, each explosive phase was counted as one cough.

### Cough recordings

**Figure 2:**
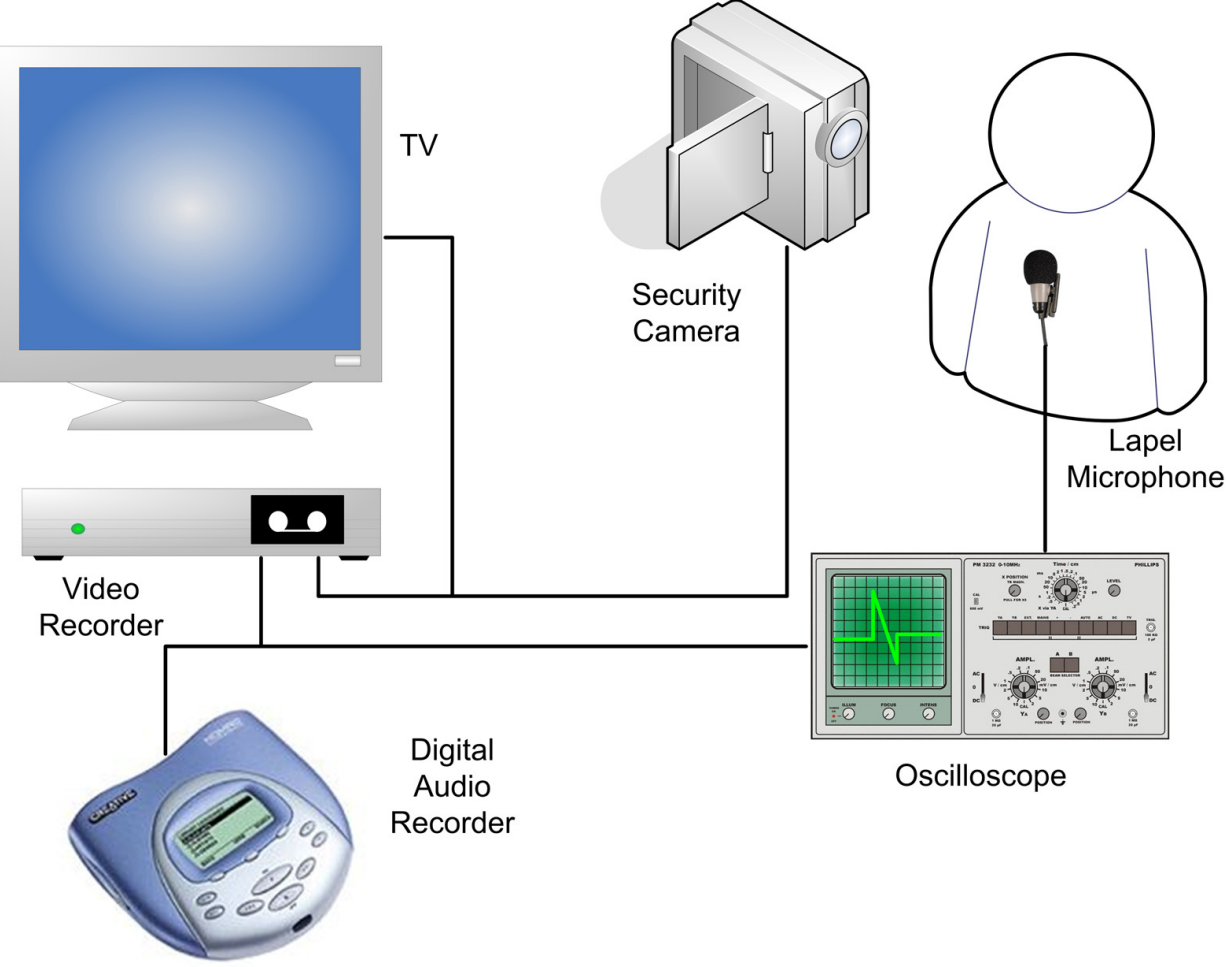
Equipment setup for simultaneous video and digital sound recordings. Note the same microphone is used to record audio into both the digital sound recorder and the video recorder. In addition to this an infra red light source is used to illuminate the subject.

The overnight recordings were made using a Nicam stereo video recorder (VC-MH713 Sharp Corporation, Osaka, Japan) and digital audio player/recorder (Creative Labs D.A.P Jukebox™, Creative Technology Ltd, Singapore). A lapel microphone (AOI ECM-1025 omni-directional electret condenser) was attached to the patient's night clothes and the signal was amplified using a pre-amplifier (BT26, B-tech International Ltd, Hong Kong). The amplified audio signal was channelled through an oscilloscope to allow real time monitoring of the signal and then to the digital recorder and the to video recorder audio input (see Figure 2).

#### Video recordings

Video recordings were made using an infrared light source (Dennard 883, Dedicated Micros Ltd, UK) and a monochrome security camera (Swann Communications, Australia). The recordings length was limited to 8 hours by the maximum length of the video tape, (4 hour video tape recorded on using long play mode). A continuous display of the time was placed above the patient's bed. The video recordings were played back in real time (i.e. over 8 hours) and explosive cough sounds counted as described above. The position in time of each cough sound was also noted so that any discrepancies between the cough counts from each device could be easily identified.

#### Digital sound recordings

The digital audio recordings were made at a sampling rate of 16 kHz, at 16 bit resolution (preset) and in wav format; this is an uncompressed sound format in common use (unlike mp3). A single 8 hour overnight recording produces a set of files that total 1.8 GB of data and can be archived on compact discs.

Explosive coughs sounds were manually counted using CoolEdit2000™ (Syntrillium, Software Corporation, Arizona). All sounds present on the digital recordings were listened to. The observer did not just listen to waveforms with the appearance of a typical cough; cough waveforms vary enormously and this would have underestimated the true number of coughs in each recording. The total number and position in time of each cough sound was noted. Using this method manual counting took 2–4 hours per overnight recording, depending on the number of coughs and other extraneous noises.

## Results

Eight subjects were studied; mean age 55 years (SD ± 11.7), 3 men, diagnoses asthma (5), rhinitis (1), gastro-oesophageal reflux disease (1) and idiopathic (1). The median cough frequency was 17.8 (IQR 5.9–28.7) cough sounds per hour in the video recordings and 17.7 (6.0–29.4) coughs per hour in the digital sound recordings (Table 1). A total of 1664 coughs were counted from the video recordings and 1684 from the audio recordings. For 5 of the 8 subjects studied slightly more coughs were counted from the digital audio recordings compared to the video recordings.

**Table 1:** Results of Cough Coughing. Total number of coughs counted for each subject from video recording and digital sound recording and subjective cough score.

**Patient ID**	**Video Cough Sounds**	**Digital Audio Cough Sounds**
1	29	29
2	164	163
3	101	105
4	2	3
5	832	842
6	161	162
7	124	121
8	251	259
**Totals**	**1664**	**1684**

**Figure 3:**
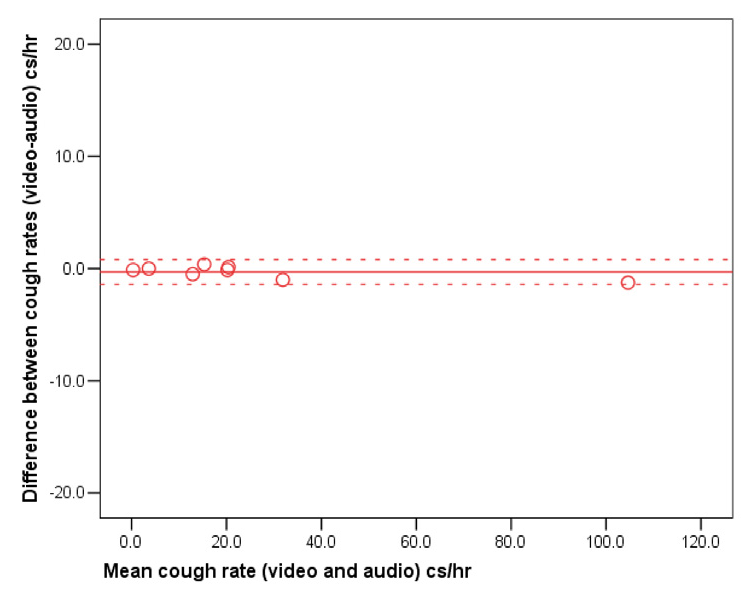
Bland Altman plot of difference between video and digital cough counts versus mean cough count. The solid horizontal red line represents the mean difference between the two methods and the red dashed lines the 95% limits of agreement.

A Bland-Altman plot (Figure 3) shows excellent agreement between the video and digital audio cough rates with a small bias towards the digital audio detecting more coughs; mean difference of -0.3 coughs per hour (SD ± 0.6). The 95% limits of agreement are -1.5 to +0.9 coughs per hour. The order in which the recordings were assessed did not significantly effect the agreement.

The cough counts for each recording technique were compared in 30 minute blocks and where a discrepancy between the counts occurred both the recordings were reviewed. The differences between the two counts appeared to be due to the differences in the sound quality of the recordings. The sound quality from the video tape was inferior leading to under counting of cough sounds especially in long peels, and occasional difficulty in distinguishing between a cough and throat clear. Overall the differences were negligible.

## Discussion

It is generally assumed that manual counting of coughs from video recordings provides the gold standard to which any automated counting system should be compared. We compared manual counting of explosive cough sounds from video with manual counting from digital audio recordings. We found excellent agreement between the two methods, with slightly more cough sounds detected from the digital audio recording. Furthermore manual cough counting from the digital sound recordings was less time consuming when compared to video.

Previous studies have used a variety of methods for objectively measuring coughing; counting coughs from video recordings [14,15] (i.e. sound and audio), sound recordings alone16 and from a combination of sound and EMG [4,5,17,18]. The quantification of cough varies in these studies with some counting explosive cough sounds16 others cough epochs [19-23] and others cough 'bouts' [24]. These are all defined in different ways by different authors as currently there is negligible standardisation or validation. This makes comparison of data between studies difficult. However, these studies do find that trained observers are able to achieve good agreement when manually counting coughs from these recordings. This is the first study to compare manual cough counting from two different sources and find excellent agreement between the cough counts.

The main limitation of this study is that the cough recordings were all performed overnight. Without a special facility to video patients during the day or confining the subjects to one room daytime video monitoring would be very difficult. We would speculate that the agreement between the video and digital audio recordings may be worse during the day as the poorer video sound quality would be more troublesome with additional speech and background noises. Additionally the cough recordings were all counted by the same individual. Although it could be argued that the agreement between the recordings may have been affected by the observer remembering the recordings when counting from the recordings from the second source, in practice, given the large amounts of data involved this seems extremely unlikely. Furthermore, the agreement in this study was slightly worse than the inter-observer agreement we had previously found (0.1 coughs per hour) [25].

Manual cough counting is extremely time-consuming and laborious, particularly from video recordings which must be reviewed in real time. It is therefore not applicable to clinical practice. Digital audio recording devices have several advantages over video. Firstly, long ambulatory recordings can be made allowing cough monitoring with unrestricted patient movement, and in their home or work environment. The performance of a cough monitor may be completely different in a subjects own environment with more background noise and movement. Secondly, counting of cough sounds is much quicker and less laborious from a digital sound recorder using audio editing software than from video. Finally, the sound quality is superior and more cough sounds can be correctly identified.

## Conclusion

Manual counting of explosive cough sounds from digital audio recordings has excellent agreement with simultaneous video recordings in laboratory conditions. As digital sound recorders have significant advantages over video recorders, ambulatory digital audio recording should now provides the gold standard for ambulatory validation of automated cough monitoring devices

## Competing interests

The author(s) declare that they have no competing interests.

## Authors' contributions

JAS recruited the subjects, performed the study and the manual counting of the video and digital sound recordings and wrote the manuscript. AW and JEE reviewed the final manuscript.
